# Casticin From Sanse Powder‐ In Vitro Release Medium Inhibits FAK/PI3K/AKT Phosphorylation Reducing Synovial Senescence and Alleviates Inflammation in Knee Osteoarthritis

**DOI:** 10.1111/jcmm.71391

**Published:** 2026-10-11

**Authors:** Jiangyu Liu, Peng Wu, Rui Jiang, Lishi Jie, Lei Shi, Houyu Fu, Yibao Wei, Deren Liu, Peimin Wang, Songjiang Yin, Jun Mao

**Affiliations:** ^1^ Department of Orthopedics The Affiliated Hospital of Nanjing University of Chinese Medicine Nanjing China; ^2^ The First College of Clinical Medicine Nanjing University of Chinese Medicine Nanjing China; ^3^ Jiangsu Provincial Engineering Research Center of TCM External Medication Development and Application Nanjing China; ^4^ Nanjing University of Chinese Medicine Clinical Research Institute for Knee Osteoarthritis Nanjing China

**Keywords:** casticin, cellular senescence, FAK/PI3K/AKT pathway, network pharmacology, Sanse Powder, transcriptomics

## Abstract

Sanse Powder (SSP) has been shown to be effective in the clinical treatment of knee osteoarthritis (KOA), although its active components and underlying mechanisms remain unclear. In this study, we collected the in vitro release medium of SSP (SSP‐IVRM) using an in vitro release test and evaluated its pharmacological effects in a KOA rat model. Ultra‐high‐performance liquid chromatography–tandem mass spectrometry (UHPLC–MS/MS) was used to identify active constituents in SSP‐IVRM, while network pharmacology and molecular docking were applied to screen key compounds and molecular targets. In vivo and in vitro experiments using histological staining and molecular biology techniques were performed to investigate the effects of casticin (CAS), a major component of SSP‐IVRM, on KOA‐related synovitis. UHPLC–MS/MS identified CAS, monocerin, and chlorogenic acid as major components. Network pharmacology and molecular docking associated these compounds with PI3K signalling and cellular senescence. CAS significantly improved synovial pathology, reduced pro‐inflammatory cytokines and chemokines, modulated fibrosis‐ and senescence‐related genes and proteins, and decreased synovial fibroblast senescence. Transcriptomic analysis further implicated cell adhesion and the PI3K–AKT pathway. Additional experiments demonstrated that CAS alleviates KOA‐related inflammation and cellular senescence by inhibiting FAK/PI3K/AKT phosphorylation. These findings indicate that CAS in SSP‐IVRM reduces synovial fibroblast senescence and inflammation through inhibition of the FAK/PI3K/AKT signalling pathway, thereby exerting therapeutic effects against KOA.

## 1 Introduction

Knee osteoarthritis (KOA), a common chronic degenerative joint disease, has shown a steadily increasing prevalence in recent years and is becoming a significant global public health challenge [1]. With the ongoing trend of global population aging, the number of KOA patients is expected to continue rising. The disease course of KOA is often accompanied by joint pain, functional impairment and reduced quality of life. Currently, there is no cure for KOA; treatment primarily relies on conservative approaches such as medication, physical therapy, and lifestyle interventions to alleviate symptoms, while severe cases may require surgical procedures like joint replacement [2, 3, 4]. However, these methods offer limited improvement in long‐term outcomes and are often associated with substantial economic and social burdens. Therefore, achieving precise treatment for KOA has become a major challenge in the field [5].

Synovial inflammation plays a critical role in the pathogenesis and progression of KOA [6]. Recent studies have shown that, although traditionally considered a degenerative disease characterized mainly by cartilage deterioration, increasing evidence indicates that synovial inflammation is also a key pathological component of KOA [7]. When the synovium of the knee joint is damaged, macrophages infiltrate the synovial tissue and release various inflammatory mediators such as interleukin‐1β (IL‐1β). The sustained high expression of these inflammatory factors not only accelerates cartilage degeneration but also promotes the secretion of matrix metalloproteinases, further exacerbating cartilage damage [8]. Additionally, synovial inflammation stimulates nerve endings, leading to joint pain [9]. As a result, precise interventions targeting synovial inflammation have become an important research direction in the comprehensive treatment of KOA and may offer new breakthroughs for future therapeutic strategies.

Sanse Powder (SSP), a novel multi‐component herbal preparation, has made significant progress in KOA treatment research in recent years [10, 11, 12]. Due to its unique pharmacological effects and safety profile, SSP has attracted growing attention. Studies have shown that topical application of SSP can exert positive therapeutic effects on KOA patients through multiple mechanisms, including inhibiting synovial inflammation and reducing cartilage destruction [10, 11, 12]. However, long‐term use of topical formulations may cause local skin damage and allergic reactions. Further identification and screening of the effective transdermal active components in SSP can optimize and refine the traditional use of SSP. By isolating its principal pharmacologically active constituents and developing more efficient modes of administration, it may be possible to enhance therapeutic outcomes and provide new treatment options and clinical choices for patients with KOA.

In this study, we prepared SSP‐ In Vitro Release Medium (SSP‐IVRM) and used UHPLC–MS/MS combined with network pharmacology to identify and screen the main active component, casticin (CAS), in SSP‐IVRM, which clears senescent synovial cells and alleviates synovial inflammation and fibrosis. Additionally, we explored the potential pharmacological mechanisms of CAS through transcriptomic analysis.

## 2 Methods and Materials

### 2.1 Materials and Reagents

DMEM high‐glucose medium (Cat# C11995500CP) and fetal bovine serum (Cat# 10099‐141) were purchased from GIBCO (USA); LPS (Cat# L2880) was obtained from Sigma‐Aldrich (USA); Casticin (Cat# B20835, ≥ 98%) was purchased from Yuanye Biotechnology (China); MIR002 (Cat# HY‐143412), Y15 (Cat# HY‐12444) and VSLRGDTRG acetate (Cat# HY‐P10941A) were obtained from MCE (China); Senescence‐Tracker Senescent Cell Fluorescent Probe (Cat# C0603), Enhanced Mitochondrial Membrane Potential Assay Kit (Cat# C2003S), Reactive Oxygen Species Assay Kit (Cat# S0035S) and Cell Counting Kit‐8 (Cat# C0038) were purchased from Beyotime Biotechnology (China); Rat CXCL1 ELISA Kit (Cat# E‐EL‐R0003), Rat CXCL3 ELISA Kit (Cat# E‐EL‐R0697), Rat IL‐6 ELISA Kit (Cat# E‐EL‐R0015) and Rat IL‐1β ELISA Kit (Cat# E‐EL‐R0012) were obtained from Elabscience (China); TGF‐β Antibody (Cat# 3711), CDC2 Antibody (Cat# 77055), FAK Antibody (Cat# 3285), PI3 Kinase p85 Antibody (Cat# 4292), AKT Antibody (Cat# 9272) and Phospho‐FAK (Tyr397) Antibody (Cat# 3283) were purchased from CST (USA); Phospho‐PI3K p85 (Tyr458)/p55 (Tyr199) Antibody (Cat# AF3242) was obtained from Affinity (China); Phospho‐AKT (Ser473) Antibody (Cat# 80455‐1‐RR), IL‐1β Antibody (Cat# 26048‐1‐AP), IL‐6 Antibody (Cat# 21865‐1‐AP), MMP3 Antibody (Cat# 17873‐1‐AP), MMP9 Antibody (Cat# 10375‐2‐AP), p53 Antibody (Cat# 10442‐1‐AP), p21 Antibody (Cat# 10355‐1‐AP) and GAPDH Antibody (Cat# 60004‐1‐Ig) were purchased from Proteintech (China).

### 2.2 Sanse Powder (SSP) and Preparation of In Vitro Release Medium for SSP

SSP was obtained from Preparation Department, Affiliated Hospital of Nanjing University of Traditional Chinese Medicine (Jiangsu pharmaceutical system number Z04000566). SSP is composed of 20 individual herbs (detailed information is shown in Table 1). All ingredients are ground into powder and mixed with Vaseline in a 1:1 ratio.

**TABLE 1:** Composition and doses of SSP.

Chinese name	Latin name	Origin	Amount (g)
Zi Jing Pi	Cercis Chinensis Cortex	*Cercis chinensis* Bunge	40
Man Jing Zi	Viticis Fructus	*Vitex trifolia* L.	40
Dang Gui	Angelicae Sinensis Radix	*Angelica sinensis* (Oliv.) Diels	10
Mu Gua	Chaenomelis Fructus	*Chaenomeles speciosa* (Sweet) Nakai	10
Zi Dan Shen	Salvia yunnanensis Radix et Rhizoma	*Salvia yunnanensis* C. H. Wright	10
Chi Shao	Paeoniae Radix Rubra	*Paeonia lactiflora* Pall.	10
Bai Zhi	Angelicae Dahuricae Radix	*Angelica dahurica* (Fisch. ex Hoffm.) Benth. et Hook.f.	10
Jiang Huang	Curcumae Longae Rhizoma	*Curcuma longa* L.	10
Du Huo	Angelicae Pubescentis Radix	*Angelica pubescens* Maxim.f. biserrata Shah et Yuan	10
Tian Hua Fen	Trichosanthis Radix	*Trichosanthes kirilowii* Maxim.	10
Chuan Niu Xi	Cyathulae Radix	*Cyathula officinalis* Kuan	10
Wei Ling Xian	Clematidis Radix et Rhizoma	*Clematis chinensis* Osbeck	10
Fang Ji	Stephaniae Tetrandrae Radix	*Stephania tetrandra* S.Moore	10
Fang Feng	Saposhnikoviae Radix	*Saposhnikovia divaricata* (Turcz.) Schischk.	10
Qiang Huo	Notopterygii Rhizoma et Radix	*Notopterygium incisum* Ting ex H. T. Chang	10
Ma Qian Zi	Strychni Semen	*Strychnos nux‐vomica* L.	10
Chuan Xiong	Chuanxiong Rhizoma	*Ligusticum chuanxiong* Hort.	4.8
Lian Qiao	Forsythiae Fructus	*Forsythia suspensa* (Thunb.) Vahl	4.8
Qin Jiao	Gentianae Macrophyllae Radix	*Gentiana macrophylla* Pall.	4.8
Gan Cao	Glycyrrhizae Radix et Rhizoma	*Glycyrrhiza uralensis* Fisch.	2.4

When preparing SSP‐IVRM using an in vitro release test (IVRT) method with a dry‐heating fully automated transdermal diffusion sampling system (SYSTEM 918‐12, LOGAN) (Figure S1A), first prepare a pH 7.4 PBS solution. Sterilize the prepared PBS by filtering it through a 0.45 μm membrane and collect it in a clean container for later use. Before the experiment, preheat the PBS buffer to 32°C to simulate human skin surface temperature. During the release study, place a measured amount of SSP (Figure S1B) into a Franz diffusion cell in the in vitro release apparatus and add an appropriate volume of preheated PBS buffer as the release medium. At predetermined time intervals, withdraw samples of the release medium, replace with an equal volume of fresh preheated PBS, and record the sampling times. At the end of the experiment, collect and store the samples (Figure S1C) for subsequent analysis.

### 2.3 Ultra‐High Performance Liquid Chromatography Tandem Mass Spectrometry (UHPLC–MS/MS)

After vacuum freeze‐drying the SSP decoction and in vitro release receiver solutions using a freeze dryer, the samples were reconstituted in 70% methanol to a final concentration of 100 mg/mL. Following centrifugation at 16,000 g for 10 min and filtration through a 0.22 μm membrane, samples were injected with three replicates per group. Mobile phase A consisted of pure water containing 0.1% formic acid, while mobile phase B was acetonitrile containing 0.1% formic acid; the elution gradient was as follows: 0–3 min, 5%–35% B; 3–6 min, 35%–55% B; 6–8 min, 55%–75% B; 8–11.5 min, 75%–95% B; 11.5–14 min, 95%–5% B. The flow rate was 0.35 mL/min, the column temperature was set to 40°C, and the injection volume was 3 μL. For mass spectrometry, the electrospray ionization (ESI) source temperature was set at 550°C; the spray voltage was 5500 V in positive ion mode and 4500 V in negative ion mode. Ion source gases I and II, and curtain gas were set at 50, 60 and 25 psi, respectively, with the collision‐induced ionization parameter set to high. QQQ scanning was performed in multiple reaction monitoring (MRM) mode, with nitrogen as the collision gas set to medium.

### 2.4 Network Pharmacology Analysis

Active ingredients of the drug identified by UHPLC–MS/MS were queried in the Traditional Chinese Medicine Systems Pharmacology Database and Analysis Platform (TCMSP) to obtain corresponding target information. The target names were then converted to the corresponding Gene IDs through the UniProt database (https://www.uniprot.org/uniprot/). Databases such as GeneCards (https://www.genecards.org/), PharmGKB (https://www.pharmgkb.org/) and the Therapeutic Targets Database (http://db.idrblab.net/ttd/) were used to retrieve target information related to ‘knee osteoarthritis.’ Target mapping was performed between the drug's active ingredient targets and the disease‐related targets to identify the intersecting target genes, and a Venn diagram was drawn. Using the STRING database (https://string‐db.org/), a ‘compound‐target’ interaction network was constructed with Cytoscape software. Core targets were identified according to the degree centrality algorithm, and the network results were visualized. R software, the pathview package and the KEGG database were used to generate a gene enrichment pathway diagram with a significance threshold of *p* < 0.01.

### 2.5 Molecular Docking

The 3D structure of AKT1, BCL2L1, BCL2, MTOR, GSK3B, CDNlA and FAK were downloaded from the PDB database (https://www.rcsb.org/), and the CAS component structure was downloaded from the PubChem database (https://pubchem.ncbi.nlm.nih.gov/). The water molecules and small molecule ligands in the key target receptor structure were removed using PyMOL2.4.1 software, and then imported into AutoDockTools1.5.6 software to select the docking site centered on the original ligand and set the gridbox. The molecular docking was completed using AutoDockVina software, and the docking results were output as txt files and pdbqt files. The minimum binding energy of the two was viewed through the txt file, and the pdbqt file was opened with PyMOL2.4.1 to view the docking image.

### 2.6 Animal Intervention and Handling

All animal experiments were approved by the Animal Ethics Committee of Nanjing University of Chinese Medicine (No. 202409A051). Sixty male Sprague–Dawley rats (6 weeks old, 250 ± 20 g) were purchased from Jiangsu Jicui Yaokang Biotechnology Co. Ltd. Using SPSS software, the 60 rats were randomly divided into six groups, with 10 rats in each group. The animals were housed at a constant temperature and humidity under a 12‐h light/dark cycle. Among them, the KOA group, low‐dose CAS group, medium‐dose CAS group, high‐dose CAS group and SSP‐IVRM‐treated group (hereafter referred to as the SSP group, consistent with the group label used in the figures) underwent anterior cruciate ligament transection (ACLT) to establish KOA rat models. Drug interventions began on Day 14 after surgery: the low‐dose, medium‐dose, and high‐dose CAS groups received intragastric administration of CAS at concentrations of 0.1 mg/kg/day, 0.2 mg/kg/day and 0.4 mg/kg/day, respectively [13, 14]; the SSP‐IVRM group received 0.5 mL SSP‐IVRM by gavage daily. All treatments were administered once daily for 28 consecutive days. The normal control group and model group were given an equivalent volume of distilled water. After the final administration, rats were fasted and deprived of water for 12 h, then anaesthetised by intraperitoneal injection of 3% sodium pentobarbital. Synovial tissue from the knee joint and peripheral blood samples were collected for subsequent analysis. Because the amount of synovial tissue available from each rat was limited and different sample‐processing procedures were required for the downstream assays, samples were allocated to different analyses. For each quantitative in vivo assay, three randomly selected rats per group were used as biologically independent replicates. Samples from different animals were not pooled. Multiple sections, microscopic fields or technical measurements obtained from the same animal were averaged to generate a single value for statistical analysis.

### 2.7 Histopathology

After fixation of synovial tissue samples with 4% paraformaldehyde, the samples were embedded in paraffin and sectioned using a microtome. Based on protocols provided by the reagent manufacturers, haematoxylin and eosin (H&E), Masson and Sirius Red staining kits were used for staining. After staining, the samples were observed under a light microscope, images were captured, and Krenn's scoring was applied to quantitatively assess synovial pathology progression.

### 2.8 Isolation and Treatment of Primary Rat Synovial Fibroblasts

Rats were euthanized with an overdose of sodium pentobarbital. Under sterile conditions, synovial tissue from the knee joint cavity was extracted. The tissue was cut into small pieces (1 mm^3^) and digested with 0.2% type I collagenase for 4 h. The digested tissue was then filtered through a 70 μm strainer, followed by centrifugation at 1200 rpm for 5 min. The resulting cells were seeded at a density of 2 × 10^5^ cells/mL in a 10 cm^2^ culture dish and incubated at 37°C with 5% CO₂.

To mimic the KOA environment, synovial fibroblasts were treated with 1 μg/mL LPS for 24 h. Based on the results of the CCK‐8 assay, CAS concentrations of 2.5, 5 and 10 μg/mL were selected as the low, medium and high concentrations, respectively, for subsequent functional assays, as none of these concentrations significantly reduced FLS viability. In experiments involving MIR002, a concentration of 1 μM was used to treat the FLS. For studies involving VSLRGDTRG acetate, a concentration of 20 μg/mL was used. When using Y‐15 to treat FLS, a concentration of 10 μM was applied.

### 2.9 Cell Counting Kit‐8 (CCK‐8)

Cells were seeded in 96‐well plates at a density of 5000 cells per well and pre‐incubated for 24 h. Cells were then treated with various concentrations of CAS (0, 2.5, 5, 10, 20 and 30 μg/mL) for 24 h. After treatment, 10 μL of CCK‐8 solution was added to each well and incubated at 37°C for 2 h. The absorbance at 450 nm was measured using a multifunctional microplate reader.

### 2.10 Western Blot (WB)

Samples were lysed using RIPA lysis buffer, followed by centrifugation at 12,000 rpm for 30 min at 4°C. Protein concentration was measured using a Nanodrop. After separation of proteins by SDS‐PAGE, proteins were transferred onto a PVDF membrane. The membrane was blocked with 5% non‐fat milk at room temperature for 2 h, then incubated overnight at 4°C with the appropriate primary antibody. The next day, the membrane was incubated with the corresponding secondary antibody at room temperature for 2 h. ECL substrate was added for chemiluminescent detection, and images were captured and saved using a gel imaging system. ImageJ software was used to analyse the bands.

### 2.11 qRT‐PCR

Total RNA was extracted from the samples using Trizol. Reverse transcription was performed with PrimeScript RT Master Mix according to the manufacturer's instructions. qRT‐PCR was carried out using SYBR Premix Ex Taq II on an ABI PRISM 7300 (Applied Biosystems, USA), following the manufacturer's protocol. Primer sequences are listed in Table 2.

**TABLE 2:** Primer sequences used in qRT‐PCR.

Target gene	Forward primer	Reverse primer
MMP3	GTTTGGCAATGCTGATGGAC	CACGCATCAAAAGGCAGTGA
MMP9	TGACAGCGACAAGAAGTGGG	CAGTGAAGCGGTACATAGGC
COL1A1	GAGCGGAGAGTACTGGATCG	GCTTCTTTTCCTTGGGGTTC
TGF‐β	CAGCAACAATTCCTGGCGATA	AGACAGCCACTCAGGCGTATC
p53	TGCTCACCCTGGCTAAAGTT	CTGAAGAGGAACCCCCAAAT
p21	CTGGTGTCTGAGCGGGAAG	GGCGTTTGGAGTGGTAGAAA
CDC2	AGGCAAGTGGAGGAAGAGGA	ATCCACAGCAGTTTTCGAGG
CCNA1	GGAGAAATGTCAGTAGCAGCA	TTCCTCTGGGAGCCAGTATG
GAPDH	GGAGAAACCTGCCAAGTATGATG	TTGCTGACAATCTTGAGGGAG

### 2.12 ELISA

Peripheral blood serum from rats and culture supernatants from primary rat synovial fibroblasts were collected, and the levels of IL‐1β, IL‐6, CXCL1 and CXCL3 were measured using ELISA kits. All procedures were carried out according to the manufacturer's instructions.

### 2.13 Immunofluorescence

Tissue sections and cell plates were fixed with 4% paraformaldehyde and permeabilized with 0.5% Triton X‐100. Non‐specific antigens were blocked using 5% bovine serum albumin. The samples were then incubated overnight with the appropriate primary antibodies. Subsequently, secondary antibodies conjugated with fluorescent probes and DAPI stain were used to label the nuclei and target proteins. After washing, cells were observed and imaged using a fluorescence microscope, and image analysis was performed with ImageJ software.

### 2.14 Mitochondrial Membrane Potential

According to the manufacturer's instructions, mitochondrial membrane potential changes were analysed using a mitochondrial membrane potential assay kit containing the JC‐1 probe. Briefly, primary rat synovial fibroblast‐like cells were seeded in 6‐well plates and treated for 24 h. The cells were then incubated with 1 mL of JC‐1 staining working solution and DAPI dye at 37°C for 30 min. After washing three times, images were observed under a fluorescence microscope, and ImageJ software was used for image analysis.

### 2.15 Reactive Oxygen Species (ROS) Detection

According to the manufacturer's instructions, a ROS detection kit containing the CM‐H2DCFDA probe was used to analyse changes in ROS levels. Briefly, primary rat synovial fibroblast‐like cells were cultured in a 6‐well plate and treated for 24 h. The cells were then incubated with 1 mL ROS staining solution and DAPI at 37°C for 30 min. After three washes, the cells were imaged under a fluorescence microscope, and ImageJ software was used for image analysis.

### 2.16 Cell Senescence Staining

According to the manufacturer's instructions, the Senescence‐Tracker probe was used to analyse β‐galactosidase activity for identifying senescent cells. Primary rat synovial fibroblast‐like cells were placed in 6‐well plates and subjected to the corresponding interventions, while Senescence‐Tracker staining working solution was added to ensure a final concentration of 10 μM in the culture medium. After the interventions and three washes, DAPI was added, and images were observed under a fluorescence microscope. ImageJ software was used for image analysis.

### 2.17 Transcriptomic Sequencing

Total RNA was extracted from primary synovial fibroblast‐like cells from each group of rats using Trizol, and the quality and quantity of RNA were assessed with a NanoDrop to ensure RNA integrity (RIN > 7.0). Sequencing libraries were constructed using the Illumina TruSeq RNA Sample Preparation Kit according to the manufacturer's instructions, and samples were sequenced on the Illumina HiSeq 4000 platform to generate 150 bp paired‐end reads. Raw sequencing data were quality controlled using FastQC and Trimomatic to remove low‐quality reads and adapter sequences. Reads were aligned to the rat reference genome using the HISAT2 tool, allowing for mismatches and splicing junctions. Transcript abundance was quantified, and principal component analysis and differential expression analysis were performed, with adjusted *p* values < 0.05 (Benjamini‐Hochberg correction) and |Log2 fold‐change (FC)| > 1. Differentially expressed genes were then subjected to functional enrichment analysis using annotation from the KEGG and GO databases. DEGs from the KOA versus Ctrl and CAS versus KOA comparisons were mapped onto the KEGG focal adhesion pathway using the Pathview package in R, with log2 fold‐change values used for colour coding.

### 2.18 Statistical Analysis

Data analysis was performed using SPSS version 22.0 and GraphPad Prism 10.0. Results are presented as the mean ± standard deviation (SD). Normality and homogeneity of variance were assessed using the Shapiro–Wilk test and Levene's test, respectively. For comparisons between two groups, an unpaired two‐tailed Student's *t*‐test was used when the data satisfied the assumptions of normality and homogeneity of variance. For comparisons among more than two groups, one‐way ANOVA followed by Tukey's multiple comparisons test was used. For experiments involving two independent factors, two‐way ANOVA followed by Šídák's multiple comparisons test was used. When the data were not normally distributed, the Mann–Whitney *U* test was used for comparisons between two groups, while the Kruskal–Wallis test followed by Dunn's multiple comparisons test was used for comparisons among more than two groups. All statistical tests were two‐sided, and *p* < 0.05 was considered statistically significant.

## 3 Results

### 3.1 SSP‐IVRM Alleviates Synovial Inflammation and Pathological Progression in KOA Rats

In previous studies, we found that SSP patch treatment could alleviate the pathological progression of KOA synovium [12]. However, the use of topical medications carries the drawback of causing local skin allergic reactions. To address this, we further developed SSP‐IVRM and investigated its pharmacological effects on KOA in in vivo experiments. ELISA was used to measure the levels of inflammatory cytokines and chemokines in the peripheral blood of rats from each group (Figure 1A). The results showed that SSP‐IVRM significantly reduced the elevated levels of IL‐1β, IL‐6, CXCL1 and CXCL3 observed during KOA progression. Histopathological analysis (Figure 1B) indicated that SSP‐IVRM decreased fibrosis and inflammatory cell infiltration in the synovial tissue of rat knee joints, resulting in lower Krenn's scores. Immunofluorescence analysis of knee joint synovial tissue (Figure 1C) demonstrated that SSP‐IVRM reduced IL‐1β levels in the synovium of KOA rats. Western blotting and RT‐PCR were used to assess the expression of fibrosis‐related proteins and genes in the synovial tissue (Figure 1D,E), revealing that SSP‐IVRM significantly decreased the protein and mRNA expression levels of MMP3, MMP9, TGF‐β and COL I in the KOA synovium.

**FIGURE 1:**
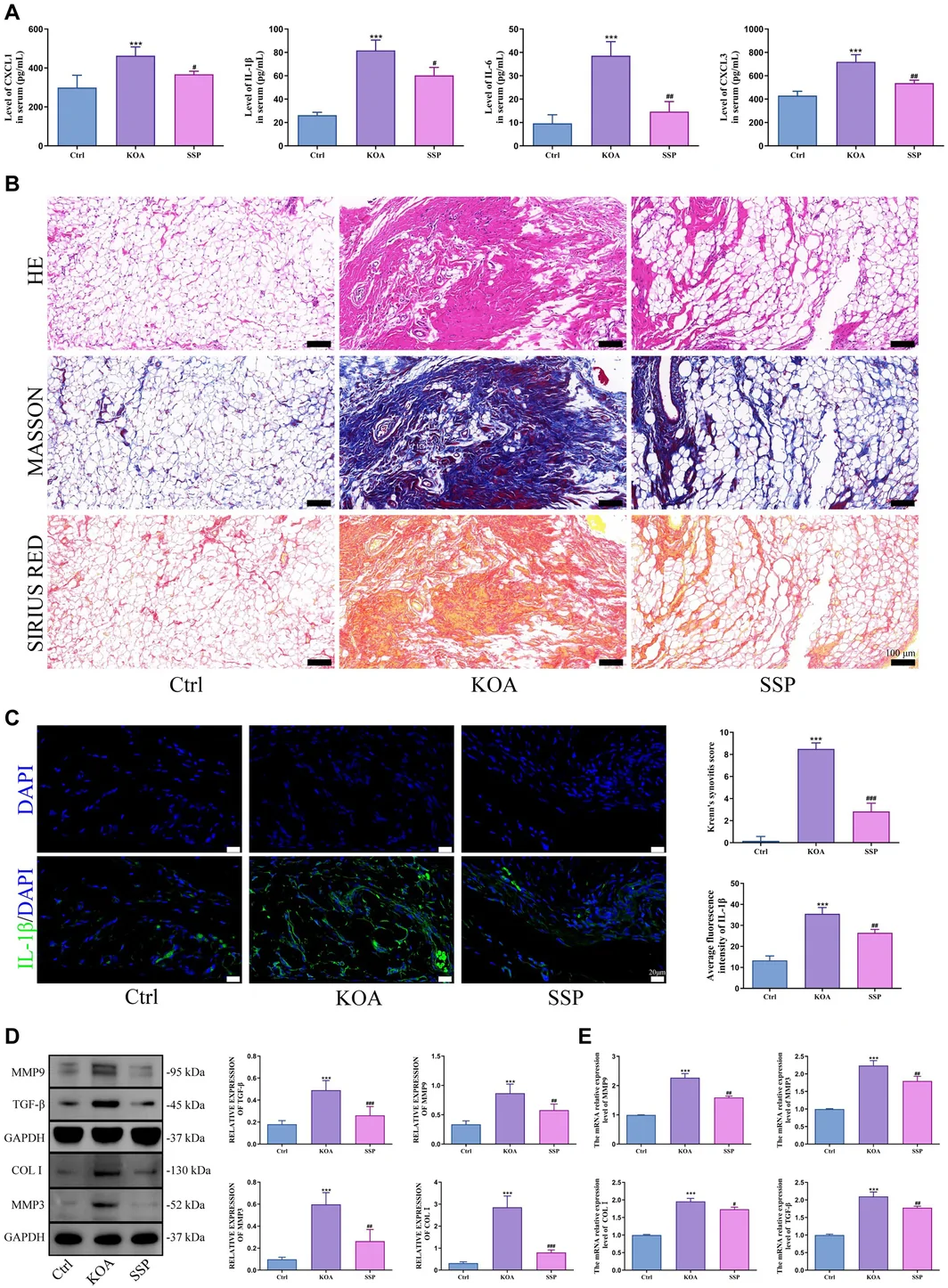
SSP‐IVRM alleviates synovial inflammation and pathological progression in KOA rats. (A) ELISA was used to detect the levels of IL‐1β, IL‐6, CXCL1 and CXCL3 in the serum of rats in each group; (B) Representative images of HE, Masson and Sirius Red staining of knee synovial tissues from each group, along with Krenn's scoring; (C) Representative images of IL‐1β immunofluorescence in knee synovial tissues from each group; (D) Western blot analysis of the protein expression levels of MMP3, MMP9, COL I and TGF‐β in the synovial tissues of rats in each group. (E) qRT‐PCR analysis of MMP3, MMP9, COL1A1 and TGF‐β mRNA expression in synovial tissues. Statistical graphs are presented as Mean ± SD (*n* = 3). *p < 0.05, **p < 0.01, ***p < 0.001 versus Ctrl group; ^#^ *p* < 0.05, ^##^ *p* < 0.01, ^###^ *p* < 0.001 versus KOA group.

### 3.2 SSP‐IVRM Components Identification and Network Pharmacology Analysis

In our in vivo experiments, we demonstrated that SSP‐IVRM exhibits anti‐inflammatory and anti‐fibrotic effects in the intervention of KOA. To further investigate which active components contribute to the efficacy of SSP‐IVRM, we performed component identification of both SSP and SSP‐IVRM using UHPLC–MS/MS to distinguish their major constituents. The total ion chromatogram (TIC) of SSP is shown in Figure 2A_1_,A_2_. A total of 172 compounds were identified in SSP (Table S1), mainly including phenolic acids, flavonoids and alkaloids. Among these, 110 compounds were detected in positive ion mode, primarily consisting of vitexin (CAS), isoorientin, cimifugin and curcumin. In negative ion mode, 62 compounds were identified, mainly including shikonin B, salvianolic acid B, chlorogenic acid and ferulic acid. The TIC of SSP‐IVRM is displayed in Figure 2B_1_,B_2_, with 54 compounds identified (Table S2), mainly consisting of flavonoids, phenolic acids and coumarins. Specifically, 33 compounds were detected in positive ion mode, primarily including CAS, isoorientin and osthole; while 21 compounds were detected in negative ion mode, mainly including chlorogenic acid, salvianolic acid B and shikonin B.

**FIGURE 2:**
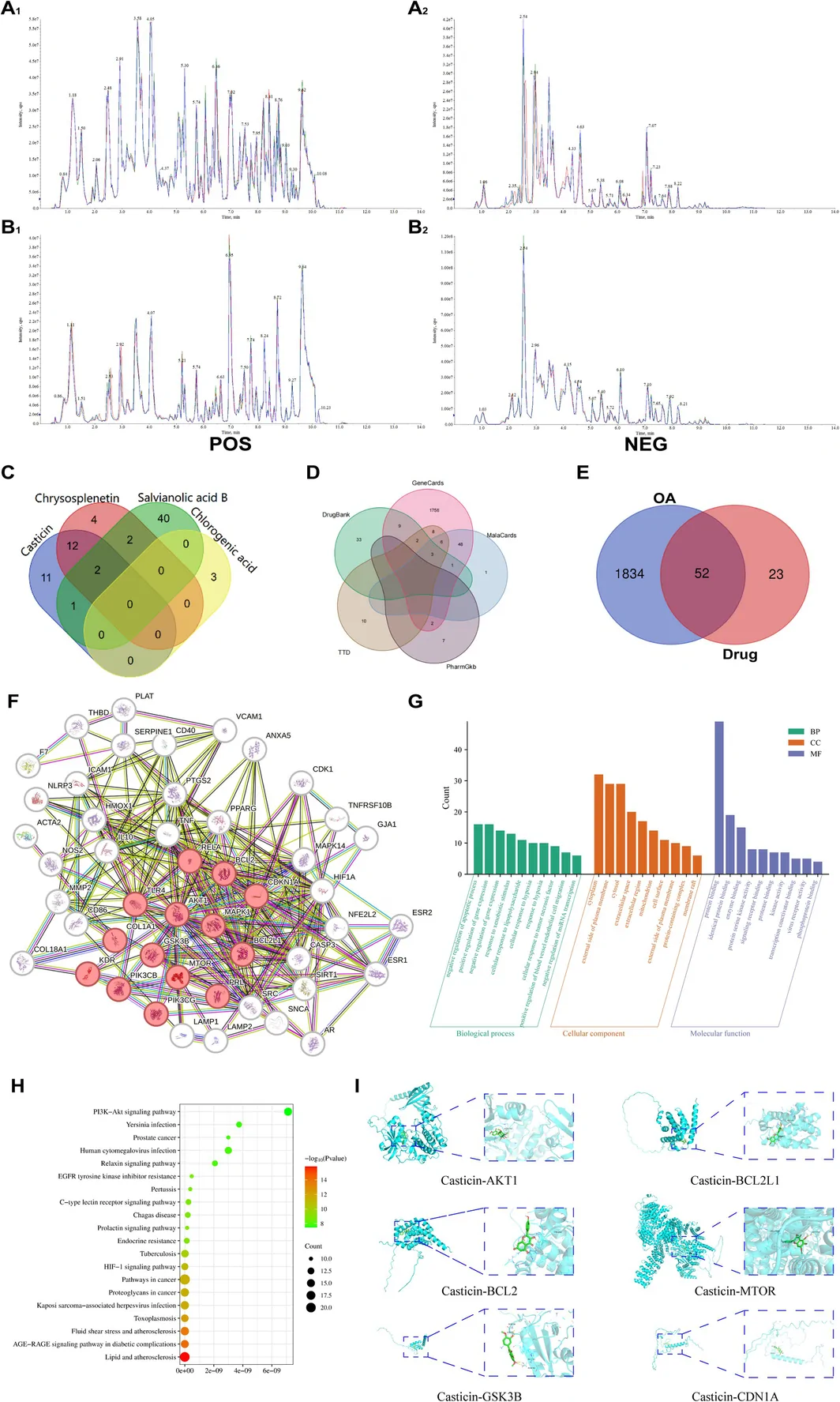
SSP‐IVRM Components Identification and Network Pharmacology Analysis. (A_1_) TIC chromatogram of SSP in positive ion mode; (A_2_) TIC chromatogram of SSP in negative ion mode; (B_1_) TIC chromatogram of SSP‐IVRM in positive ion mode; (B_2_) TIC chromatogram of SSP‐IVRM in negative ion mode; (C) Drug target analysis of CAS, isoorientin, chlorogenic acid and salvianolic acid B; (D) Analysis of OA disease targets from the GeneCards, DrugBank, MalaCards, PharmGKB and TTD databases; (E) Venn diagram showing the intersection between OA disease targets and drug targets; (F) PPI network illustrating the overlap between OA disease targets and drug targets; (G) GO enrichment analysis of genes at the intersection of OA disease targets and drug targets; (H) KEGG enrichment analysis of genes at the intersection of OA disease targets and drug targets; (I) Molecular docking of CAS with AKT1, BCL2L1, BCL2, MTOR, GSK3B and CDNlA.

We conducted network pharmacology analysis on the main active components of SSP‐IVRM, namely, CAS, Isoorientin, chlorogenic acid and salvianolic acid B, which ranked among the top four in SSP‐IVRM (Figure 2C). The results showed that CAS had 26 drug targets, Isoorientin had 20 drug targets, chlorogenic acid had 3 drug targets and salvianolic acid B had 45 drug targets. After removing duplicate entries, we obtained a total of 75 unique drug targets. At the same time, osteoarthritis (OA)‐related targets were collected from the GeneCard, DrugBank, MalaCards, PharmGKB and TTD databases. After eliminating duplicates, 1886 OA‐related targets were identified (Figure 2D). By overlapping the 75 drug targets with the 1886 OA‐related targets, 52 drug‐disease targets were obtained (Figure 2E). Using the STRING database, we screened these targets and constructed a PPI network for the 52 potential targets, identifying 14 genes related to the PI3K‐Akt signalling pathway (Figure 2F). Gene Ontology (GO) enrichment analysis of the main active components of SSP‐IVRM showed that their targets were mainly involved in protein binding and cytoplasm, among other terms (Figure 2G). Based on KEGG pathway enrichment analysis for the main targets of CAS, the primary active components of SSP‐IVRM were found to be associated with lipid and atherosclerosis and the PI3K‐Akt signalling pathway (Figure 2H). Further molecular docking between CAS and the targets on the PI3K‐Akt signalling pathway revealed that CAS had strong affinity with AKT1, BCL2L1, BCL2, MTOR, GSK3B and CDNlA (Figure 2I). Western blot and RT‐PCR were used to assess the protein and gene expression levels of fibrosis‐related markers in synovial tissue (Figure 1D,E), showing that CAS was able to reduce both the protein and mRNA expression levels of MMP3, MMP9, TGF‐β and COL I in the synovial tissue.

### 3.3 CAS Alleviates Synovial Inflammation and Cellular Senescence in KOA Rats

To further investigate the pharmacodynamic effects of CAS, the main active component of SSP‐IVRM, and to validate the findings from network pharmacology, we conducted in vivo experiments to observe the effects of different concentrations of CAS on KOA rats. Histopathological results (Figure 3A) showed that CAS intervention reduced fibrosis and inflammatory cell infiltration in the synovial tissue of rat knee joints, decreased the Krenn's score, and demonstrated a concentration‐dependent drug effect. ELISA assays assessed the levels of inflammatory and chemotactic factors in the peripheral blood of rats in each group (Figure 3B), revealing that various concentrations of CAS reduced the KOA‐induced elevations of IL‐1β, IL‐6, CXCL1 and CXCL3. Immunofluorescence results (Figure 3C) indicated that CAS reduced the protein expression of IL‐1β in synovial tissue in a dose‐dependent manner. In line with the network pharmacology results, we further examined p21 CDNlA, a key gene closely associated with cell cycle and senescence. Using Western Blot and RT‐PCR, we measured the expression levels of senescence‐related proteins and genes (p53, p21CDNlA, CCNA1 and CDC2) as well as fibrosis‐related markers in the synovial tissue (Figure 3D,E). Various concentrations of CAS reduced the protein and mRNA expression levels of MMP3, MMP9, TGF‐β and COL I in KOA synovial tissue, decreased the protein and mRNA expression levels of the cell senescence markers p53 and p21CDNlA, and upregulated the protein and mRNA expression levels of the cell cycle proteins CDC2 and CCNA1.

**FIGURE 3:**
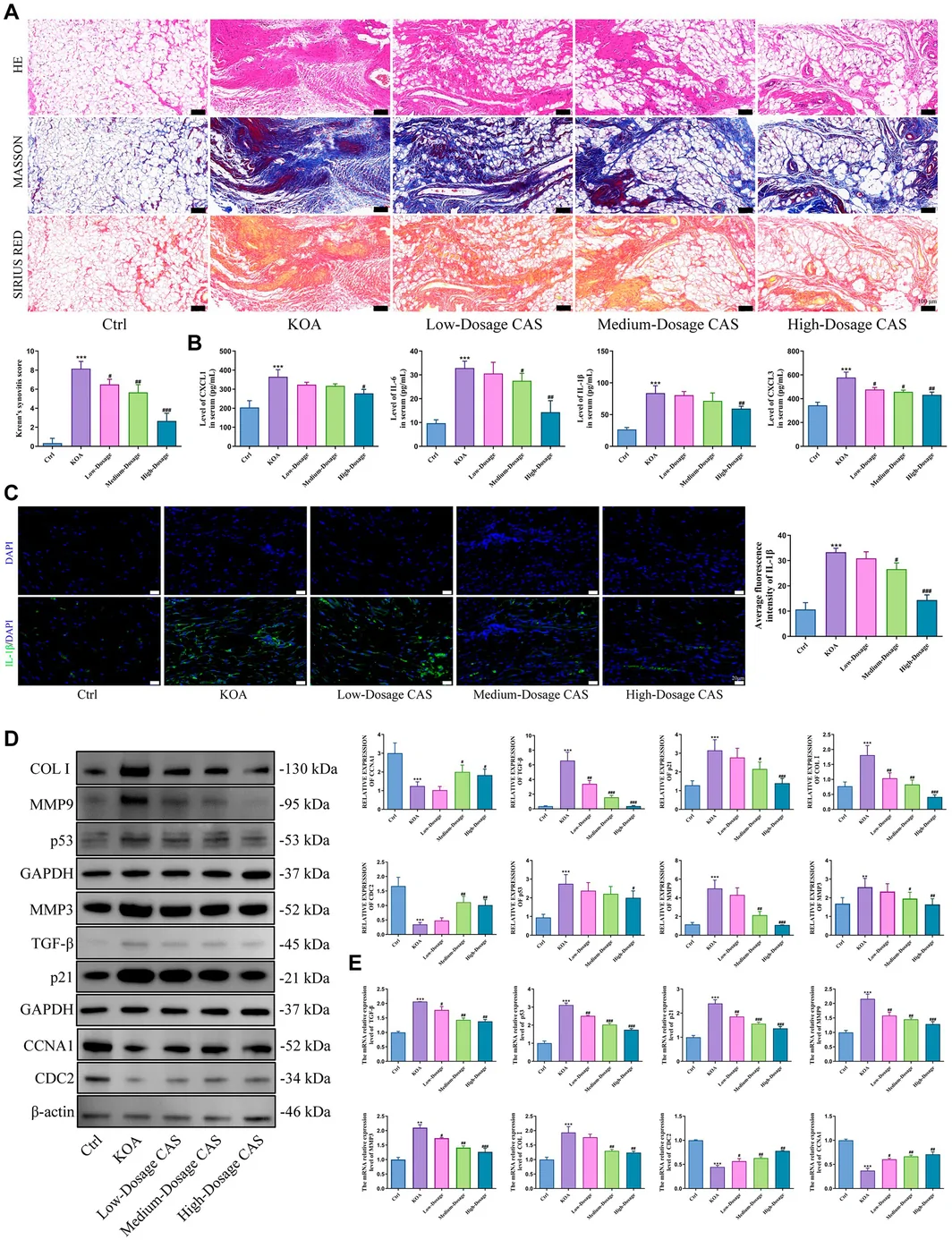
CAS alleviates synovial inflammation and cellular senescence in KOA rats. (A) Representative images of HE, Masson and Sirius Red staining of synovial tissue from rat knee joints in each group, along with Krenn's scores; (B) Serum levels of IL‐1β, IL‐6, CXCL1 and CXCL3 in each group of rats as measured by ELISA; (C) Representative immunofluorescence images of IL‐1β in synovial tissue from rat knee joints in each group; (D) Protein expression levels of MMP3, MMP9, COL I, TGF‐β, p53, p21, CDC2 and CCNA1 in synovial tissue from each rat group as determined by Western Blot. (E) qRT‐PCR analysis of the mRNA expression levels of MMP3, MMP9, COL1A1, TGF‐β, p53, p21/CDKN1A, CDC2 and CCNA1 in synovial tissues from each rat group. Statistical graphs are presented as Mean ± SD (*n* = 3). *p < 0.05, **p < 0.01, ***p < 0.001 versus Ctrl group; ^#^ *p* < 0.05, ^##^ *p* < 0.01, ^###^ *p* < 0.001 versus KOA group.

### 3.4 CAS Improves Inflammation and Cellular Senescence in LPS‐Induced Rat Synovial Fibroblast‐Like Cells

In the in vitro experiments, we first used the CCK‐8 assay to assess the cytotoxic effects of CAS on primary rat synovial fibroblast‐like cells (FLS) (Figure 4A). We found that CAS at 20 and 30 μg/mL significantly reduced cell viability, whereas 2.5, 5 and 10 μg/mL did not significantly affect cell viability. Therefore, 2.5, 5 and 10 μg/mL were selected for the subsequent dose–response experiments. To further investigate the effect of CAS on FLS under an LPS‐induced KOA‐like environment, we used ELISA to measure the levels of inflammatory and chemotactic factors in the FLS culture supernatant (Figure 4B). The results showed that CAS at various concentrations reduced the LPS‐induced upregulation of IL‐1β, IL‐6, CXCL1 and CXCL3 in a dose‐dependent manner. Immunofluorescence analysis of IL‐1β and IL‐6 in FLS treated with different concentrations of CAS (Figure 4C) yielded results consistent with the ELISA findings. Furthermore, western blotting was performed to assess the expression of proteins related to cellular senescence and fibrosis in FLS (Figure 4D). CAS was able to decrease the expression of MMP3, MMP9, TGF‐β and COL I induced by LPS stimulation, as well as the senescence markers p53 and p21 CDKN1A. Conversely, CAS increased the expression of cell cycle proteins CDC2 and CCNA1. To further evaluate the effect of CAS on cellular senescence, we used reactive oxygen species (ROS) probes, mitochondrial membrane potential assays and senescence‐associated staining to observe FLS after CAS treatment (Figure 4E–G). The results showed that LPS treatment significantly increased ROS levels in FLS, decreased mitochondrial membrane potential and led to mitochondrial dysfunction, thereby accelerating FLS senescence. However, CAS intervention reduced LPS‐induced ROS accumulation, mitigated mitochondrial damage, increased mitochondrial membrane potential and decreased the percentage of senescence‐positive cells.

**FIGURE 4:**
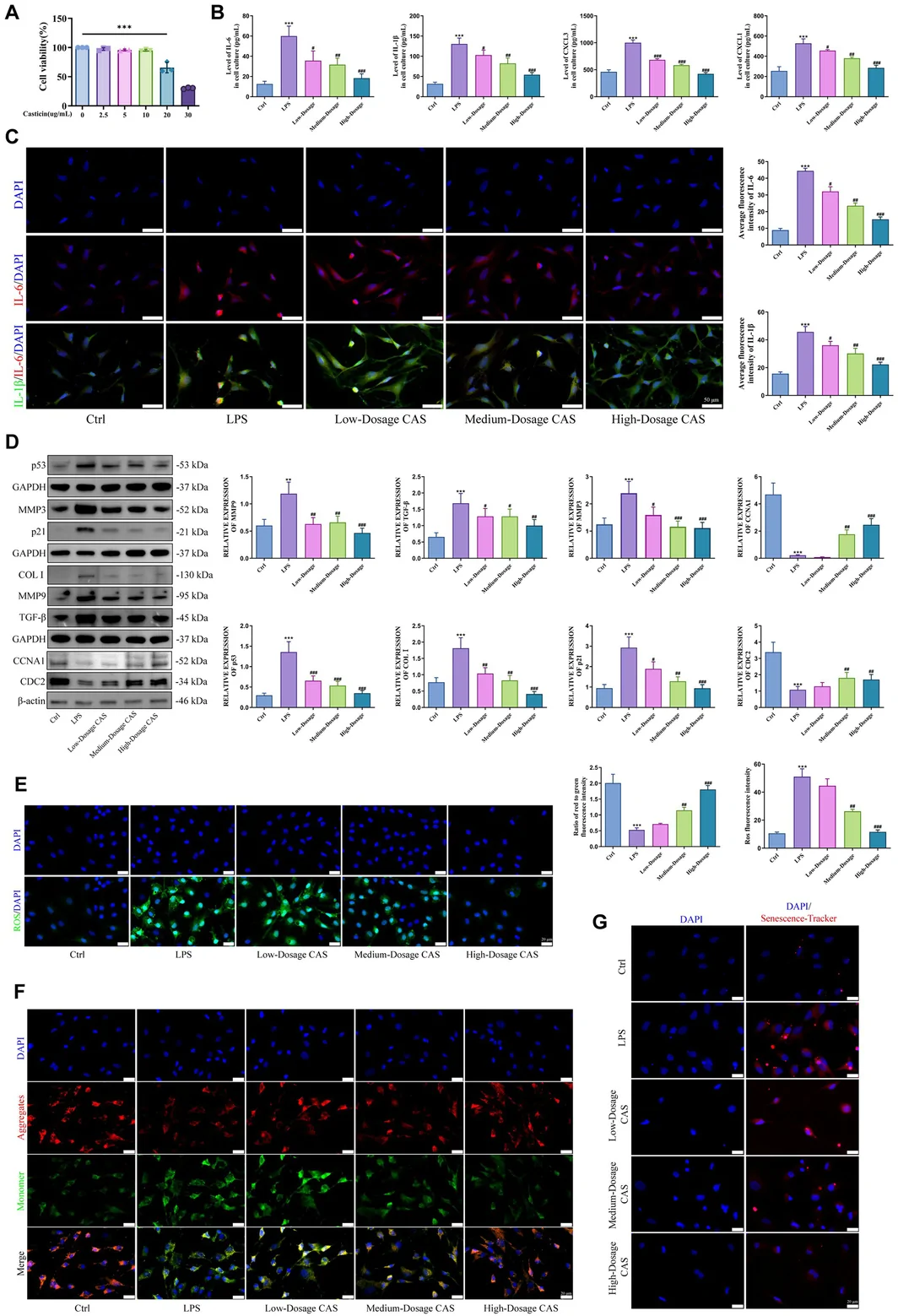
CAS improves inflammation and cellular senescence in LPS‐induced rat synovial fibroblast‐like cells. (A) CCK‐8 assay to evaluate the cytotoxic effects of CAS on FLS; (B) ELISA to measure the levels of IL‐1β, IL‐6, CXCL1 and CXCL3 in the culture media of rat FLS from each group; (C) Representative immunofluorescence images of IL‐1β and IL‐6 in FLS from each group; (D) Western blot analysis of protein expression levels of MMP3, MMP9, COL I, TGF‐β, p53, p21, CDC2 and CCNA1 in FLS from each group; (E) CM‐H2DCFDA probe to assess ROS levels in FLS from each group; (F) JC‐1 probe to detect mitochondrial membrane potential levels in FLS from each group; (G) Representative images of senescent cells labelled by Senescence‐Tracker probe in FLS from each group. Statistical graphs are presented as Mean ± SD (*n* = 3). *p < 0.05, **p < 0.01, ***p < 0.001 versus Ctrl group; ^#^ *p* < 0.05, ^##^ *p* < 0.01, ^###^ *p* < 0.001 versus LPS group.

### 3.5 CAS Reduces the Inflammatory State of Rat Synovial Fibroblast‐Like Cells by Lowering Cellular Senescence Levels

To investigate the role of CAS in improving cellular senescence and inflammation, we used MIR002 to promote the expression of p21 CDN1A, thereby simulating cell cycle arrest and inducing a senescent state in cells. CAS was used at 10 μg/mL in these experiments, representing the highest tested concentration that did not significantly affect FLS viability. We assessed the status of FLS after MIR002 and CAS interventions using ROS probes, mitochondrial membrane potential assays and cell senescence probes (Figure 5A–C). The results indicated that MIR002 treatment further increased ROS levels in FLS, decreased mitochondrial membrane potential, caused mitochondrial dysfunction and promoted LPS‐induced senescence in FLS. In contrast, CAS intervention attenuated the effects of MIR002 by reducing ROS accumulation in FLS, repairing mitochondrial dysfunction, increasing mitochondrial membrane potential and decreasing the proportion of senescence‐positive cells. Correspondingly, Western blot analysis (Figure 5D) revealed that MIR002 upregulated the expression of fibrotic markers MMP3, MMP9, TGF‐β, COL I and senescence‐related proteins p53 and p21 CDN1A in FLS, while downregulating cell cycle proteins CDC2 and CCNA1. CAS intervention reversed the effects of MIR002, reducing the expression of MMP3, MMP9, TGF‐β, COL I, p53 and p21 CDN1A, while increasing CDC2 and CCNA1 protein levels. In addition, we used immunofluorescence and ELISA to assess the levels of inflammatory and chemotactic factors in FLS (Figure 5E,F). The results showed that MIR002 increased the levels of inflammatory and chemotactic factors IL‐1β, IL‐6, CXCL1 and CXCL3 in FLS, whereas CAS intervention counteracted MIR002's effects by reducing these factors' levels.

**FIGURE 5:**
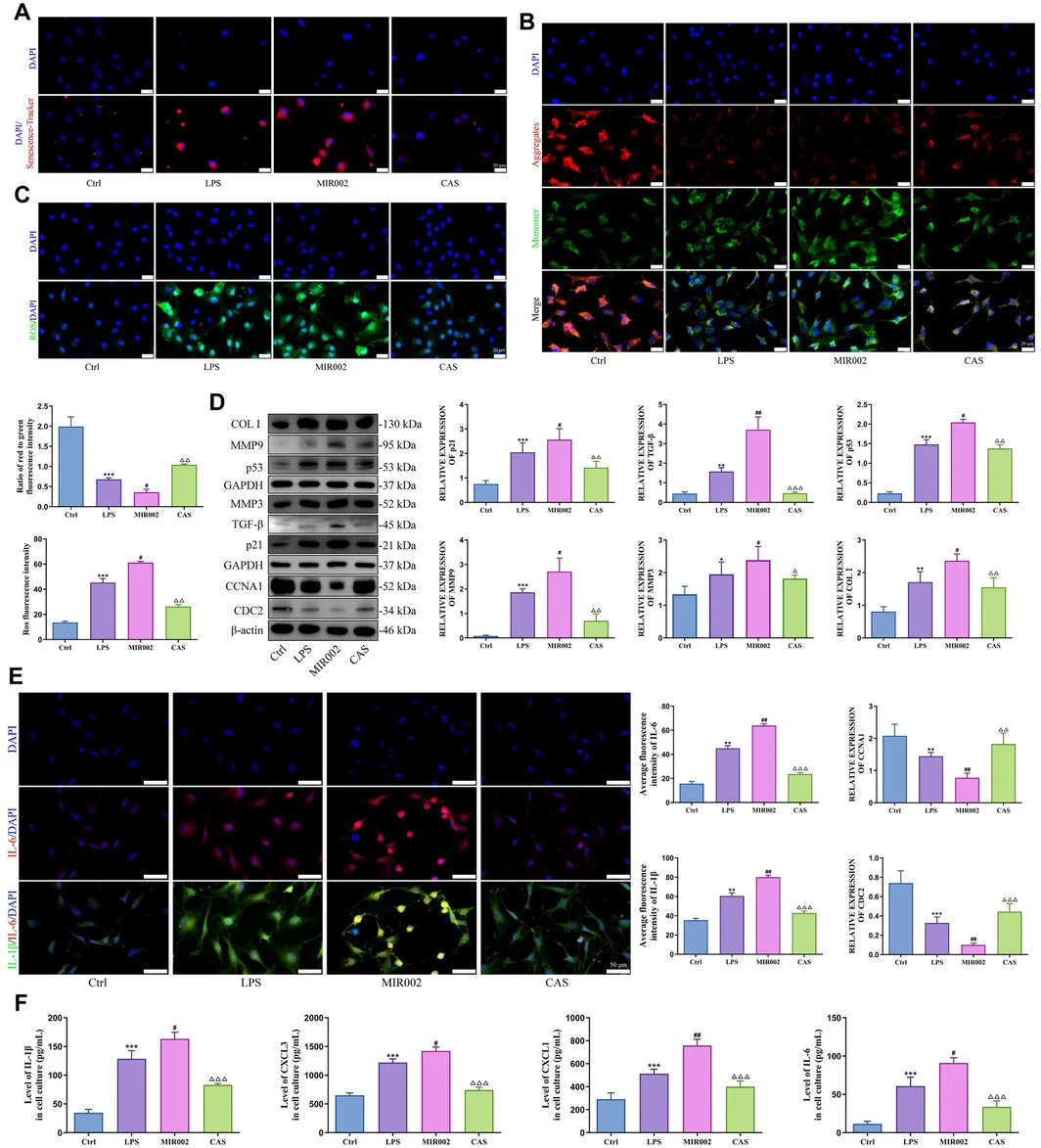
CAS reduces the inflammatory state of rat synovial fibroblast‐like cells by lowering cellular senescence levels. (A) Representative images of senescent positive cells in rat FLSs from each group detected using the Senescence‐Tracker probe; (B) Mitochondrial membrane potential levels in rat FLSs from each group measured by the JC‐1 probe; (C) Intracellular ROS levels in rat FLSs from each group assessed with the CM‐H2DCFDA probe; (D) Protein expression levels of MMP3, MMP9, COL I, TGF‐β, p53, p21, CDC2 and CCNA1 in rat FLSs from each group detected by Western blotting (WB); (E) Representative immunofluorescence images of IL‐1β and IL‐6 in rat FLSs from each group; (F) Levels of IL‐1β, IL‐6, CXCL1 and CXCL3 in the culture supernatant of rat FLSs from each group measured by ELISA. Statistical graphs are presented as Mean ± SD (*n* = 3). *p < 0.05, **p < 0.01, ***p < 0.001 versus Ctrl group; ^#^ *p* < 0.05, ^##^ *p* < 0.01, ^###^ *p* < 0.001 versus LPS group; ^△^ *p* < 0.05, ^△△^ *p* < 0.01, ^△△△^ *p* < 0.001 versus MIR002 group.

### 3.6 Transcriptome Sequencing Suggests That CAS May Function Through Focal Adhesion and the PI3K‐AKT Signalling Pathway

We further investigated the potential drug targets and pharmacological mechanisms of CAS using transcriptomic sequencing. First, we used correlation heatmaps and principal component analysis to visualize the relationships among FLS in each group (Figure 6A,B). The results showed that FLS treated with CAS were more similar to the Ctrl group, while FLS subjected to LPS intervention (to simulate the KOA environment) showed significant genetic differences compared to the other two groups. Venn diagram analysis was used to compare the number of genes among the FLS groups (Figure 6C). Further, volcano plots and bar graphs revealed that, during the KOA process, 3921 genes were upregulated and 1904 genes were downregulated in FLS (Figure 6D); following CAS intervention, 2045 genes were upregulated and 5058 genes were downregulated in FLS (Figure 6E). Hierarchical clustering heatmaps displayed the distribution of differentially expressed genes among the three FLS groups (Figure 6F). GO enrichment analysis of differentially expressed genes between the CAS and KOA groups (Figure 6H) indicated that, in terms of biological processes (BP), CAS intervention was mainly associated with protein phosphorylation, obsolete oxidation–reduction process, phosphorylation, DNA damage response, cell adhesion and other terms. In terms of cellular components (CC) and molecular functions (MF), the results aligned with those of network pharmacology, and were also related to mitochondrion and ATP binding. KEGG enrichment analysis of differential genes between the KOA and Ctrl groups showed that KOA was primarily associated with metabolic pathways, ECM‐receptor interaction, focal adhesion and PI3K‐Akt signalling pathway. KEGG enrichment analysis of differential genes between the CAS and KOA groups showed significant enrichment in cell adhesion, inflammatory factors, fibrosis and cellular senescence, including focal adhesion, ECM‐receptor interaction, PI3K‐Akt signalling pathway, cell adhesion molecules, TNF signalling pathway, p53 signalling pathway and TGF‐beta signalling pathway. To further examine focal adhesion‐related transcriptional changes, DEGs from the KOA versus control (Ctrl) and CAS versus KOA comparisons were mapped onto the KEGG focal adhesion pathway (Figure S3). In the KOA versus Ctrl comparison, DEGs mapped to PKC, PI3K, PIP5K, ROCK, B‐Raf and cIAPs were predominantly upregulated, whereas Fyn was downregulated. In the CAS versus KOA comparison, DEGs mapped to these nodes and to the FAK and PAK nodes were predominantly downregulated, suggesting that CAS partially reversed KOA‐associated transcriptional changes. These findings support the involvement of focal adhesion signalling in the effects of CAS and provide a transcriptomic basis for the subsequent functional evaluation of the FAK/PI3K/AKT signalling axis.

**FIGURE 6:**
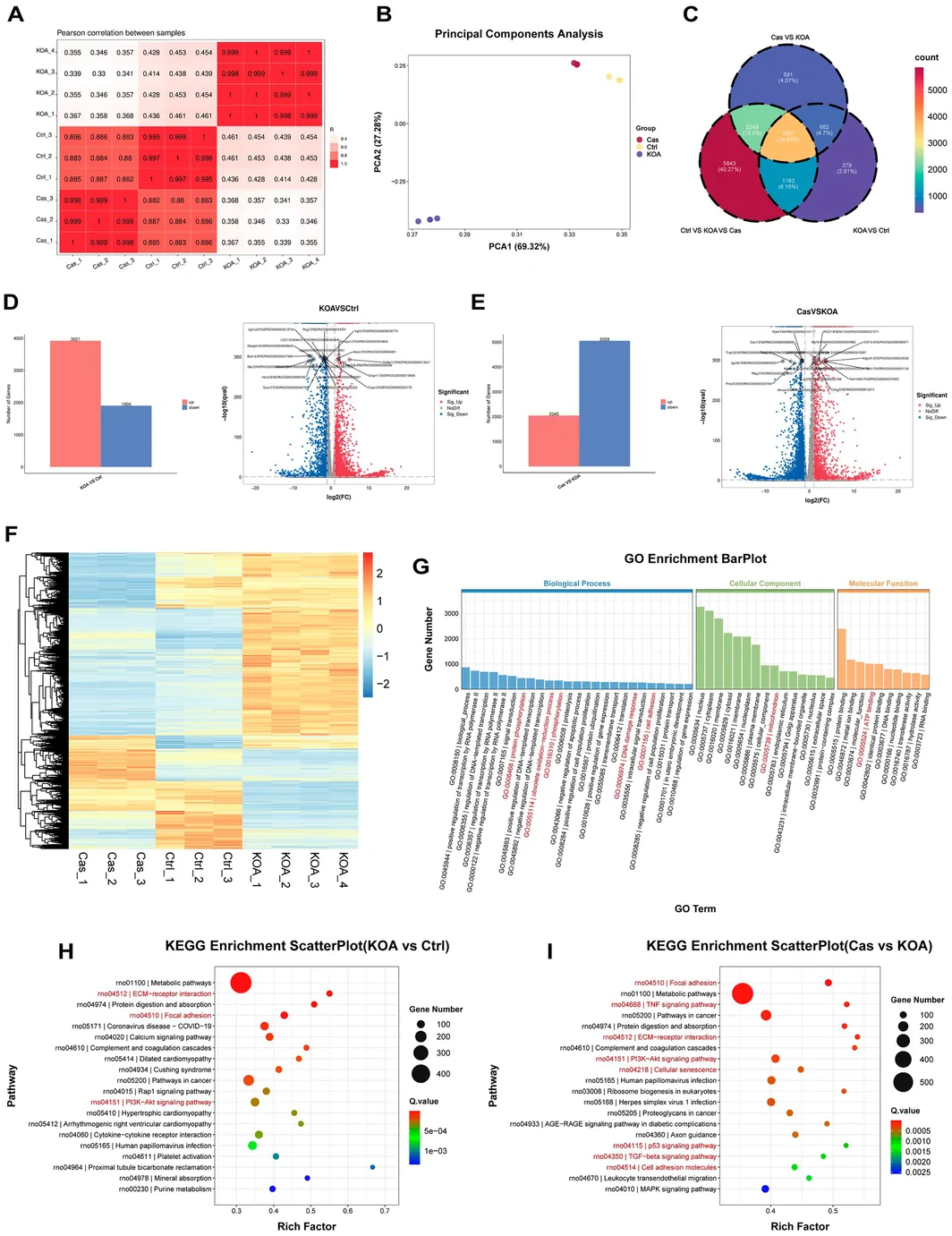
Transcriptome sequencing suggests that CAS may function through focal adhesion and the PI3K‐AKT signalling pathway. (A) Sample correlation heatmap of FLSs in each group of rats; (B) Principal component analysis of FLSs in each group; (C) Venn diagram showing the number of differentially expressed genes among groups; (D) Bar chart and volcano plot illustrating differentially expressed genes between KOA and Ctrl group FLSs; (E) Bar chart and volcano plot illustrating differentially expressed genes between CAS and KOA group FLSs; (F) Cluster analysis heatmap of FLSs in each group; (G) GO enrichment analysis of differentially expressed genes between CAS and KOA group FLSs; (H) KEGG enrichment analysis of differentially expressed genes between KOA and Ctrl group FLSs; (I) KEGG enrichment analysis of differentially expressed genes between CAS and KOA group FLSs.

### 3.7 CAS Inhibits FAK/PI3K/AKT Phosphorylation Process to Reduce Synovial Cell Senescence and Improve Synovial Inflammation

Based on the results of GO enrichment analysis and KEGG enrichment analysis from transcriptome sequencing, we believe that CAS plays a role in improving synovial inflammation and cellular senescence through processes related to protein phosphorylation and the FAK‐PI3K‐AKT signalling axis. Therefore, we used Western blotting to detect the expression levels of proteins involved in the FAK‐PI3K‐AKT signalling axis in synovial tissue and FLS (Figure 7A,B). The results showed that during KOA progression in synovial tissue and in LPS‐induced FLS, the ratios of p‐FAK/FAK, p‐PI3K/PI3K and p‐AKT/AKT were significantly increased, indicating increased phosphorylation of FAK, PI3K and AKT. In contrast, CAS intervention significantly reduced these ratios, indicating that CAS inhibited the phosphorylation of FAK, PI3K and AKT. Further immunofluorescence analysis of p‐FAK and p‐PI3K fluorescence intensity in FLS (Figure 7C) yielded results consistent with those of the Western blot, indicating that CAS markedly lowers the protein expression levels of p‐FAK and p‐PI3K induced by LPS. To further investigate the potential molecular interaction between CAS and FAK, additional molecular docking was performed. The best‐ranked CAS–FAK docking pose yielded a predicted binding affinity of −8.4 kcal/mol. CAS formed conventional hydrogen bonds with Glu430 and Thr503 at distances of 2.5 and 2.9 Å, respectively, together with multiple van der Waals, alkyl and π–sigma interactions within the FAK kinase‐domain binding pocket (Figure S2). These findings support a potential interaction between CAS and FAK but do not independently establish direct binding or inhibition of FAK kinase activity.

**FIGURE 7:**
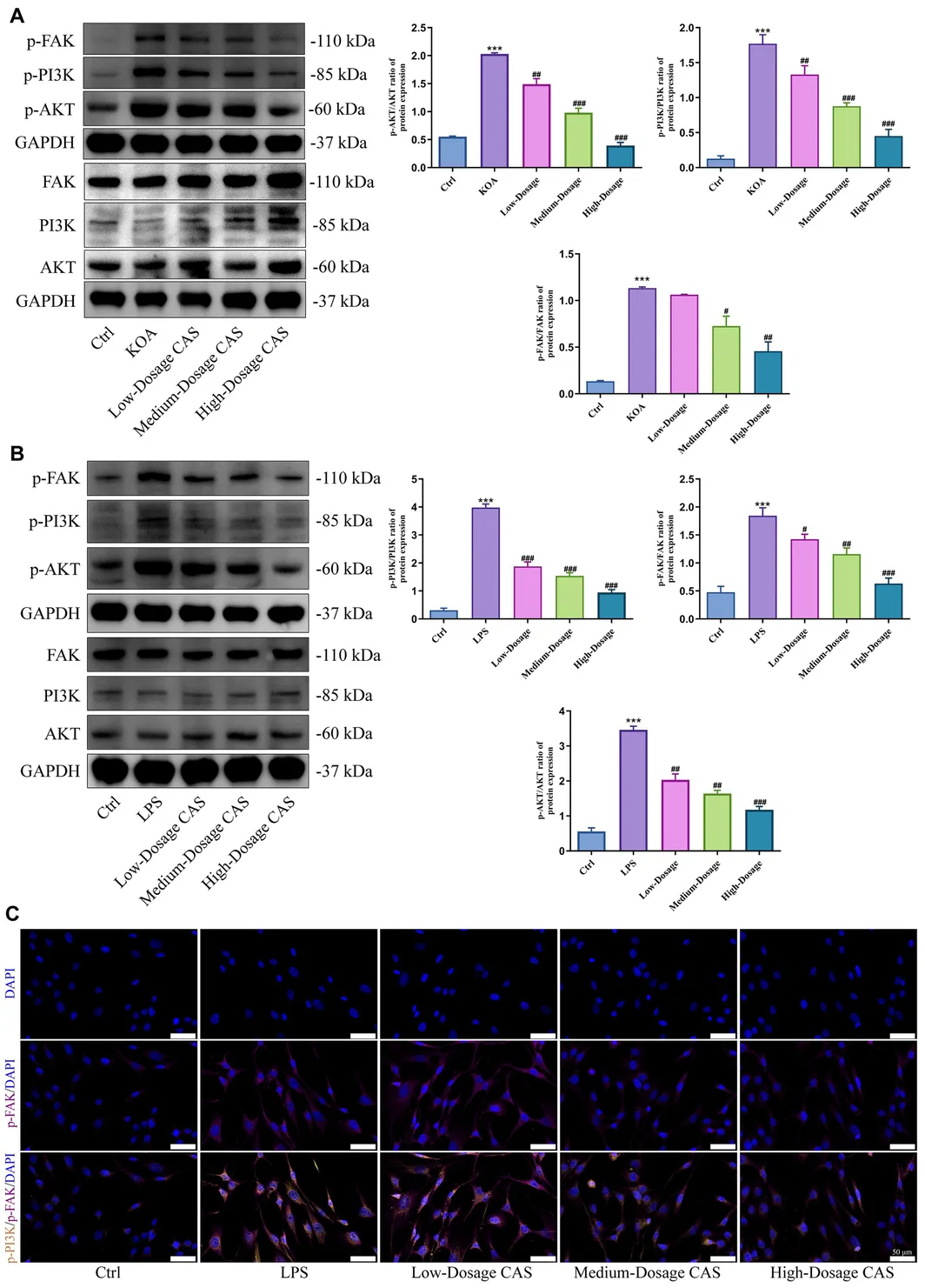
CAS inhibits FAK/PI3K/AKT phosphorylation in both in vivo and in vitro experiments. (A) WB analysis of protein expression levels of p‐FAK, FAK, p‐PI3K, PI3K, p‐AKT and AKT in synovial tissue from each group of rats; (B) WB analysis of protein expression levels of p‐FAK, FAK, p‐PI3K, PI3K, p‐AKT and AKT in FLS from each group of rats; (C) Representative immunofluorescence images of p‐FAK and p‐PI3K in FLS from each group of rats. Statistical graphs are presented as Mean ± SD (*n* = 3). *p < 0.05, **p < 0.01, ***p < 0.001 versus Ctrl group; ^#^ *p* < 0.05, ^##^ *p* < 0.01, ^###^ *p* < 0.001 versus LPS group.

To investigate the role of the FAK‐PI3K‐AKT signalling axis in the regulation of synovial cell senescence and inflammation by CAS, we utilized VSLRGDTRG to intervene in FLS to maintain FAK phosphorylation levels and Y‐15 to inhibit FAK phosphorylation, thereby further assessing the function of CAS within the FAK‐PI3K‐AKT pathway. CAS was used at 10 μg/mL in the mechanistic intervention experiments shown in Figure 8. First, we examined the fluorescence intensities of p‐FAK and p‐PI3K in FLS from each group using immunofluorescence (Figure 8A). Following VSLRGDTRG treatment, the effect of CAS was attenuated, with increased fluorescence intensities of p‐FAK and p‐PI3K; conversely, Y‐15 led to further reductions in the fluorescence intensities of p‐FAK and p‐PI3K. In parallel, Western blot analysis (Figure 8B,C) indicated that VSLRGDTRG weakened the dephosphorylation effect of CAS, resulting in upregulation of the ratios of p‐FAK/FAK, p‐PI3K/PI3K and p‐AKT/AKT, indicating increased phosphorylation of FAK, PI3K and AKT. In contrast, the combination of CAS and Y‐15 further decreased these ratios, indicating further inhibition of FAK, PI3K and AKT phosphorylation. Simultaneously, VSLRGDTRG increased the protein expression levels of MMP9, TGF‐β, COL I, p53 and p21 CDNlA, while downregulating the expression of CDC2 and CCNA1; Y‐15 produced the opposite effects, decreasing MMP9, TGF‐β, COL I, p53 and p21 CDNlA expression and increasing CDC2 and CCNA1 protein levels. Additionally, in assays related to FLS cellular senescence (Figure 8D–F), we found that VSLRGDTRG diminished the effect of CAS, elevating ROS levels in FLS, reducing mitochondrial membrane potential, causing mitochondrial dysfunction and promoting FLS senescence. In contrast, Y‐15 enhanced the effect of CAS, reducing ROS accumulation, increasing mitochondrial membrane potential, ameliorating mitochondrial dysfunction and decreasing the proportion of senescence‐positive FLS cells. Finally, using ELISA to measure the levels of inflammatory and chemotactic factors in FLS (Figure 8G), results showed that VSLRGDTRG could weaken the anti‐inflammatory effects of CAS, leading to increased levels of IL‐1β, IL‐6, CXCL1 and CXCL3. Conversely, Y‐15 augmented the effects of CAS, further reducing the levels of IL‐1β, IL‐6, CXCL1 and CXCL3 in FLS.

**FIGURE 8:**
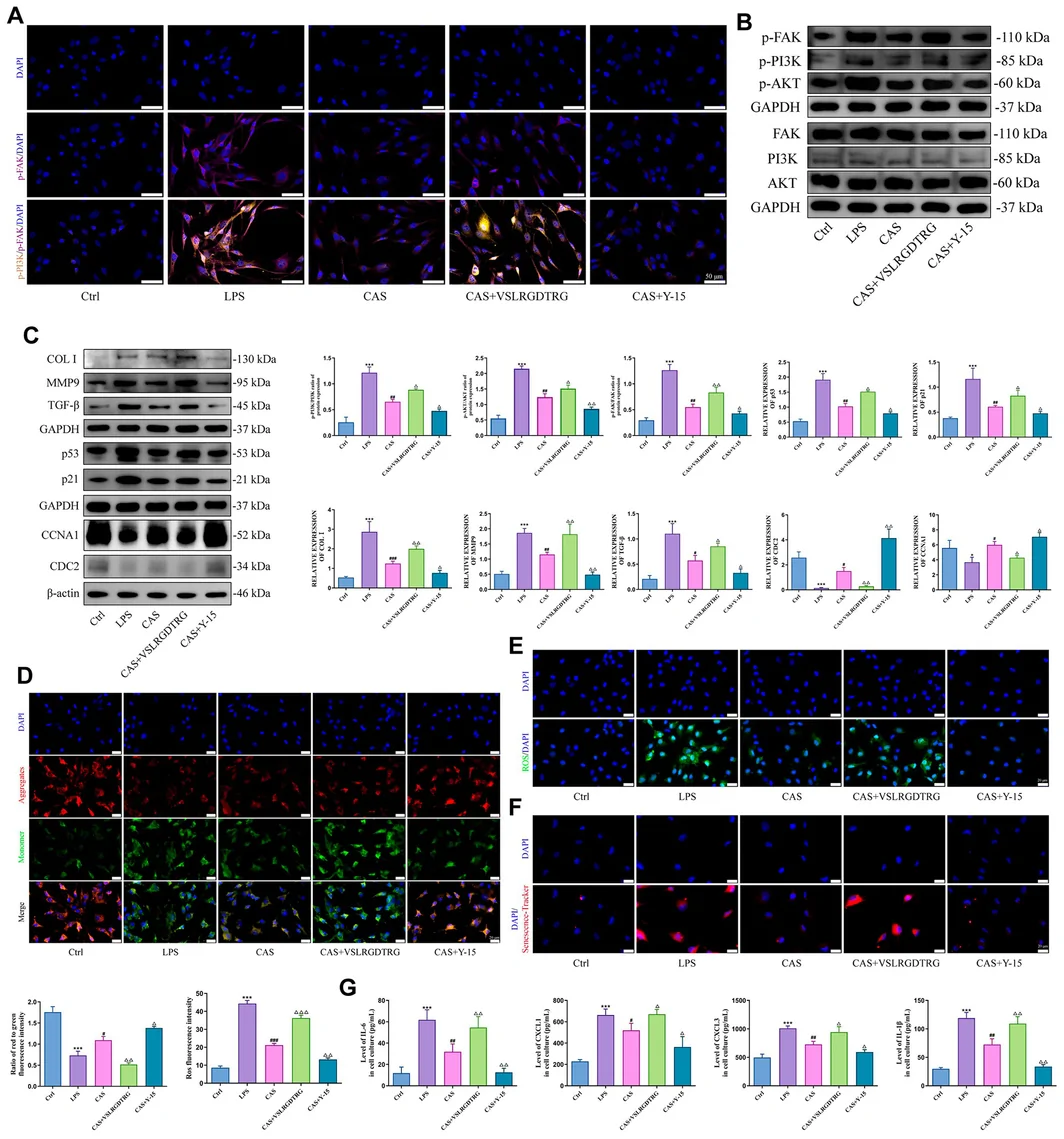
CAS inhibits FAK/PI3K/AKT phosphorylation process to reduce synovial cell senescence and improve synovial inflammation. (A) Representative immunofluorescence images of p‐FAK and p‐PI3K in FLS from each rat group; (B) Western blot analysis of protein expression levels of p‐FAK, FAK, p‐PI3K, PI3K, p‐AKT and AKT in FLS from each group; (C) Western blot analysis of protein expression levels of MMP9, COL I, TGF‐β, p53, p21, CDC2 and CCNA1 in FLS from each group; (D) Assessment of mitochondrial membrane potential in FLS from each group using the JC‐1 probe; (E) Detection of ROS levels in FLS from each group using the CM‐H2DCFDA probe; (F) Representative images of senescent FLS detected with the Senescence‐Tracker probe in each group; (G) ELISA analysis of IL‐1β, IL‐6, CXCL1 and CXCL3 levels in the culture medium of FLS from each group. Statistical graphs are presented as Mean ± SD (*n* = 3). *p < 0.05, **p < 0.01, ***p < 0.001 versus Ctrl group; ^#^ *p* < 0.05, ^##^ *p* < 0.01, ^###^ *p* < 0.001 versus LPS group; ^△^ *p* < 0.05, ^△△^ *p* < 0.01, ^△△△^ *p* < 0.001 versus CAS group.

## 4 Discussion

Knee osteoarthritis (KOA) is now recognized as a complex disease involving multiple tissues—such as cartilage, synovium, bone and ligaments—and the interplay of various factors [8]. Beyond the traditional focus on cartilage degeneration and osteophyte formation, the regulation of synovial inflammation has become a major area of research in KOA [15]. The synovium lines the inner surface of the knee joint capsule and plays an essential role in joint function. It secretes synovial fluid, which lubricates and nourishes the articular cartilage, maintains the homeostasis of the joint environment, serves as a barrier and participates in immune regulation to prevent harmful substances from entering the joint [16]. Therefore, synovial inflammation is not only one of the key pathological changes in the early stages of KOA but also persists throughout disease progression [17]. Inflammation of the synovium leads to the accumulation of inflammatory cells and mediators, promotes synovial cell proliferation, angiogenesis and joint effusion, and accelerates cartilage degradation and subchondral bone damage, thereby worsening joint pain and dysfunction [18]. In addition, chronic synovial inflammation can trigger synovial fibrosis, resulting in joint stiffness and limited mobility [19]. Recent studies suggest that synovial inflammation is not merely a secondary manifestation of KOA but may also play a role in disease initiation and drive the entire process of cartilage degeneration [19]. Therefore, effective control of synovial inflammation is one of the key strategies for precise intervention and prevention of KOA.

It is well known that traditional Chinese herbal medicine has a long history and demonstrates significant clinical efficacy in treating diseases [20]. Numerous experimental and clinical studies have shown that herbs such as Tripterygium wilfordii, Scutellaria baicalensis and *Curcuma longa* are clearly effective in the treatment of synovial inflammation. These herbs can alleviate synovial inflammatory responses and cellular proliferation by inhibiting the expression of inflammatory factors and regulating signalling pathways such as NF‐κB and MAPK [21, 22]. In our previous work, external application of SSP was found to reduce pathological progression in the synovial tissue of KOA and decrease synovial inflammation [12]. However, there are still several limitations and shortcomings to this method of external application. Further optimizing the mode of drug intervention could enhance therapeutic efficacy and reduce adverse reactions in clinical practice [23]. Therefore, in this study, we prepared a transdermal release receiving solution of SSP, aiming to explore and optimize its administration method and further enhance the therapeutic effect of SSP in the treatment of KOA.

The complexity of components in traditional Chinese herbal medicines and the unclear mechanisms of action have hindered their clinical application [24]. Identifying the active ingredients in these herbs and clarifying their specific therapeutic mechanisms are major challenges faced by researchers today. With advancements in genomics, proteomics, systems biology, mathematics and computer science, the integration of multi‐omics technologies now enables comprehensive and complementary analysis of multidimensional data [25]. Network pharmacology can predict the regulatory networks of multiple components, multiple targets and multiple pathways in traditional Chinese medicine, while transcriptomics provides an accurate reflection of gene expression changes and biological responses [26]. The combination of these approaches not only helps to uncover the complex synergistic mechanisms of traditional Chinese medicine, but also facilitates the screening and validation of key therapeutic targets and biomarkers, thereby improving the accuracy and depth of mechanistic studies [27]. In this study, UHPLC–MS/MS was used to investigate the active components in the transdermal release receptor solution of SSP, and further exploration of the potential molecular mechanisms by which SSP treats KOA synovial inflammation was conducted based on analyses using network pharmacology and transcriptomics, including enrichment analysis of shared targets, identification of key targets and selection of relevant signalling pathways. CAS, as a major component in the SSP transdermal release receptor solution, received particular attention. As a flavonoid compound, CAS has been found to act on the free radical scavenging system, reduce lipid peroxidation, enhance the activities of endogenous antioxidant enzymes such as SOD and CAT and decrease oxidative stress‐induced cellular damage. Additionally, in models of inflammatory diseases such as pneumonia and encephalitis, CAS has been shown to inhibit the production of inflammatory cytokines including TNF‐α, IL‐6 and IL‐1β, and to block activation of the NF‐κB signalling pathway, thereby exerting significant anti‐inflammatory effects.

With advancing age, prolonged mechanical overload, and inflammatory stimuli, synovial fibroblasts are prone to cellular senescence [28]. Senescent cells no longer proliferate but secrete large amounts of inflammatory factors, proteases and pro‐fibrotic molecules—a phenomenon known as the senescence‐associated secretory phenotype (SASP) [29]. SASP factors not only amplify local inflammation in the synovium but also promote the deposition of collagen and matrix proteins, accelerating synovial fibrosis [30]. Additionally, impaired clearance of senescent cells leads to persistent inflammation and abnormal tissue remodelling. Increasing evidence suggests that synovial cell senescence is a key driver of sustained and chronic synovial inflammation and its progression to fibrosis, playing a crucial role in the pathogenesis of KOA [31]. Our research indicates that CAS inhibits the p53 signalling pathway in cells, thereby reducing cellular senescence. We believe that the antioxidant function of CAS plays a vital role in mitigating cellular senescence by decreasing the accumulation of reactive oxygen species (ROS) and repairing mitochondrial damage. This, in turn, slows the progression of cellular senescence and reduces SASP secretion, ultimately lowering inflammation and fibrosis.

Focal adhesions are signalling integration and transduction platforms formed by the binding of transmembrane proteins, such as integrins, to the extracellular matrix, which are then linked to the intracellular actin cytoskeleton through a series of anchoring proteins [32]. Focal adhesions not only maintain cell morphology but also regulate cell proliferation, migration and survival by activating multiple signalling pathways, including PI3K/AKT and MAPK [33]. Changes in the extracellular environment or mechanical stress are sensed by integrins, which activate signalling molecules like FAK, thereby modulating intracellular stress pathways [34]. Abnormalities in focal adhesion structure or aberrant activation of mechanotransduction can induce oxidative stress responses, DNA damage and cell cycle arrest, subsequently activating senescence‐associated molecules such as p53 and p21, and driving cells into a senescent state [35, 36]. In this study, we investigated the role of the FAK/PI3K/AKT signalling pathway in the process of FLS senescence. We propose that external risk factors activate the FAK/PI3K/AKT pathway, leading to phosphorylation modifications and increased accumulation of ROS within cells, which in turn impairs mitochondrial function and accelerates FLS cellular senescence. Moreover, CAS mitigates the inflammatory state of FLS by inhibiting activation of the FAK/PI3K/AKT pathway, thereby exerting antioxidant and anti‐senescence effects.

However, this study still has certain limitations. Although molecular docking suggested a potential interaction between CAS and FAK, direct CAS–FAK binding and inhibition of FAK kinase activity have not been experimentally established, and an indirect effect mediated through upstream integrin–FAK signalling cannot be excluded. Moreover, because SSP‐IVRM and CAS were administered by gavage, transdermal efficacy, comparative oral–transdermal pharmacokinetics and long‐term safety remain to be evaluated. In future studies, we plan to include validation using different animal models and human‐derived samples to enhance the clinical relevance and translatability of our results. We will also strengthen systematic assessments of pharmacokinetics, safety and long‐term efficacy to provide more comprehensive data for clinical applications. In terms of molecular mechanism studies, we intend to further explore the interaction networks between the FAK/PI3K/AKT pathway and other signalling pathways, and clarify the roles of key molecules using gene editing and other approaches.

## 5 Conclusion

In summary, by identifying the main active component, CAS, in SSP‐IVRM using UHPLC–MS/MS and predicting its drug targets through network pharmacology and molecular docking techniques, we found that CAS can reduce synovial inflammation and fibrosis in KOA rats, as well as decrease cellular senescence in synovial tissue. Further transcriptomic analysis was conducted to explore the potential drug mechanisms of CAS, and experimental validation showed that CAS may exert its effects by inhibiting cellular senescence in synovial tissue via suppression of the FAK‐PI3K‐AKT signalling axis, thereby improving synovial inflammation and fibrosis in KOA. Overall, CAS improves synovial inflammation and fibrosis in KOA by inhibiting FAK phosphorylation and clearing senescent cells.

## Author Contributions

**Jiangyu Liu:** writing – original draft. **Peng Wu:** writing – original draft, funding acquisition. **Rui Jiang:** investigation. **Lishi Jie:** data curation. **Lei Shi:** methodology. **Houyu Fu:** resources. **Yibao Wei:** data curation. **Deren Liu:** project administration. **Peimin Wang:** project administration, funding acquisition. **Songjiang Yin:** writing – review and editing. **Jun Mao:** writing – review and editing, funding acquisition.

## Funding

This work was supported by the National Natural Science Foundation of China (No. 82205143); Special Program for Outstanding Young PhD Training, Jiangsu Provincial Hospital of Traditional Chinese Medicine (No. 2023QB0122); Third Batch of High‐level Academic Talent, Jiangsu Provincial Hospital of Traditional Chinese Medicine (No. y2021rc20); Fourth Batch of Outstanding Clinical Talent in Traditional Chinese Medicine, Jiangsu Province; Key Project of the Jiangsu Provincial Traditional Chinese Medicine Science and Technology Development Plan (ZD202213); Jiangsu Provincial Medical Key Discipline (Laboratory) Cultivation Unit (JSDW202252); Clinical Medical Innovation Center for Knee Osteoarthritis of TCM, Jiangsu Provincial Hospital of Chinese Medicine (Y2023zx05); Knee Osteoarthritis Specialized Clinical Research Institute, Nanjing University of Chinese Medicine (LCZBYJYZZ2024‐003).

## Disclosure

Animal Welfare: The present study followed international, national and/or institutional guidelines for humane animal treatment and complied with relevant legislation.

## Ethics Statement

Ethical approval for this study was obtained from the Institutional Animal Care and Use Committee at Nanjing University of Chinese Medicine (No. 202409A051).

## Consent

Data was de‐identified after collection and therefore there was no consent required from individual patients according to the ethical approval.

## Conflicts of Interest

The authors declare no conflicts of interest.

## Supporting information


**Figure S1:** Preparation of Sanse Powder‐ in vitro release medium (SSP‐IVRM). (A) Dry‐heating, fully automated transdermal diffusion sampling system (LOGAN SYSTEM 918–12) used for the in vitro release test. (B) SSP topical plaster preparation used for the test. (C) Representative SSP‐IVRM sample collected after the in vitro release test.

**Figure S2:** Molecular docking analysis of casticin with focal adhesion kinase (FAK). From left to right, the overall docking pose of casticin within FAK, an enlarged view of the binding pocket, and a two‐dimensional interaction map are shown. Casticin formed conventional hydrogen bonds with Glu430 and Thr503 at distances of 2.5 and 2.9 Å, respectively, together with additional van der Waals, alkyl, and π–sigma interactions with surrounding residues. The best predicted binding affinity was −8.4 kcal/mol.

**Figure S3:** KEGG pathway mapping of focal adhesion‐related differentially expressed genes in KOA and after CAS treatment. (A) KOA versus control (Ctrl). (B) CAS versus KOA. Red and green indicate higher and lower expression, respectively, in the first group relative to the second group in each comparison. Colour intensity represents the magnitude of the log2 fold change. CAS, casticin; Ctrl, control; KOA, knee osteoarthritis.

**Table S1:** Identification of major chemical constituents in Sanse Powder (SSP). The table presents the compound identity and classification, molecular formula, Q1 and Q3 ions, molecular weight, ionization mode, identification level, CAS number, compound ID, and signal intensities in three SSP samples. Q1 and Q3 denote the precursor and product ions, respectively.

**Table S2:** Identification of major chemical constituents in Sanse Powder‐ in vitro release medium (SSP‐IVRM). The table presents the compound identity and classification, molecular formula, Q1 and Q3 ions, molecular weight, ionization mode, identification level, CAS number, compound ID, and signal intensities in three SSP‐IVRM samples. Q1 and Q3 denote the precursor and product ions, respectively.

## Data Availability

All results and data are kept in the section for Departments of Orthopedics, the Affiliated Hospital of Nanjing University of Chinese Medicine, Nanjing, China. These will be made available from the corresponding author on reasonable request.
