# Supplementary material for: Casticin From Sanse Powder‐ In Vitro Release Medium Inhibits FAK/PI3K/AKT Phosphorylation Reducing Synovial Senescence and Alleviates Inflammation in Knee Osteoarthritis
Source: J Cell Mol Med. 2026 Oct 11;30(19):e71391. doi: 10.1111/jcmm.71391 (PMC13655228; doi:10.1111/jcmm.71391)
Supplement: Supplementary file 1 [file JCMM-30-e71391-s003.docx]

**Supplementary Figure S1: Preparation of Sanse Powder in vitro release medium (SSP-IVRM).**

(A) Dry-heating, fully automated transdermal diffusion sampling system (LOGAN SYSTEM 918-12) used for the in vitro release test. (B) SSP topical plaster preparation used for the test. (C) Representative SSP-IVRM sample collected after the in vitro release test.
