# Supplementary material for: Casticin From Sanse Powder‐ In Vitro Release Medium Inhibits FAK/PI3K/AKT Phosphorylation Reducing Synovial Senescence and Alleviates Inflammation in Knee Osteoarthritis
Source: J Cell Mol Med. 2026 Oct 11;30(19):e71391. doi: 10.1111/jcmm.71391 (PMC13655228; doi:10.1111/jcmm.71391)
Supplement: Supplementary file 2 [file JCMM-30-e71391-s004.doc]

**Supplementary Figure S2. Molecular docking analysis of casticin with focal adhesion kinase (FAK).**

From left to right, the overall docking pose of casticin within FAK, an enlarged view of the binding pocket, and a two-dimensional interaction map are shown. Casticin formed conventional hydrogen bonds with Glu430 and Thr503 at distances of 2.5 and 2.9 Å, respectively, together with additional van der Waals, alkyl, and π–sigma interactions with surrounding residues. The best predicted binding affinity was −8.4 kcal/mol.
