# Supplementary material for: Casticin From Sanse Powder‐ In Vitro Release Medium Inhibits FAK/PI3K/AKT Phosphorylation Reducing Synovial Senescence and Alleviates Inflammation in Knee Osteoarthritis
Source: J Cell Mol Med. 2026 Oct 11;30(19):e71391. doi: 10.1111/jcmm.71391 (PMC13655228; doi:10.1111/jcmm.71391)
Supplement: Supplementary file 3 [file JCMM-30-e71391-s005.doc]

**Supplementary Figure S3. KEGG pathway mapping of focal adhesion-related differentially expressed genes in KOA and after CAS treatment.**

(A) KOA versus control (Ctrl). (B) CAS versus KOA. Red and green indicate higher and lower expression, respectively, in the first group relative to the second group in each comparison. Color intensity represents the magnitude of the log2 fold change. CAS, casticin; Ctrl, control; KOA, knee osteoarthritis.
